# Persistent deterioration of visuospatial performance in spaceflight

**DOI:** 10.1038/s41598-021-88938-6

**Published:** 2021-05-05

**Authors:** Endre Takács, Irén Barkaszi, István Czigler, Lívia Gabriella Pató, Anna Altbäcker, Joseph McIntyre, Guy Cheron, László Balázs

**Affiliations:** 1 Institute of Cognitive Neuroscience and Psychology, ELKH Research Centre for Natural Sciences, Budapest, 1117 Hungary; 2 Institute of Psychology, Eötvös Loránd University, Budapest, 1075 Hungary; 3 Doctoral School of Psychology, Eötvös Loránd University, Budapest, 1075 Hungary; 4 Centre de Neurophysique, Physiology, Et Pathology, Centre National de La Recherche Scientifique, Université Paris Descartes, 75270 Paris Cedex 06, France; 5 Health Division, Tecnalia Research and Innovation, 20009 Donostia-San Sebastian, Spain; 6 Ikerbasque Science Foundation, 48009 Bilbao, Spain; 7 Laboratory of Neurophysiology Movement Biomechanics, ULB Neuroscience Institute, Université Libre de Bruxelles, 1070 Anderlecht, Belgium; 8 Laboratory of Electrophysiology, Université de Mons, 7000 Mons, Belgium

**Keywords:** Neuroscience, Cognitive neuroscience

## Abstract

Although human adaptation to spaceflight has been studied for decades, little is known about its long-term effects on brain and behavior. The present study investigated visuospatial performance and associated electrophysiological responses in astronauts before, during, and after an approximately half-year long mission to the International Space Station. Here we report findings demonstrating that cognitive performance can suffer marked decrements during spaceflight. Astronauts were slower and more error-prone on orbit than on Earth, while event-related brain potentials reflected diminished attentional resources. Our study is the first to provide evidence for impaired performance during both the initial (~ 8 days) and later (~ 50 days) stages of spaceflight, without any signs of adaptation. Results indicate restricted adaptability to spaceflight conditions and calls for new research prior to deep space explorations.

## Introduction

The manned space mission to reach Mars is such a complex endeavor that cannot be successful without the constant peak performance of astronauts. However, the integrity of the human cognitive system is endangered by both direct effects of microgravity and indirect effects associated with the spaceflight environment. Due to the lack of hydrostatic pressure, an important direct effect of microgravity is the headward fluid shift^1^. It is thought to be at least partly responsible for neuro-ophthalmological symptoms, including increased intracranial pressure and visual problems, constituting the so-called Spaceflight-Associated Neuro-Ocular Syndrome (SANS)^2^. Microgravity directly impacts the neurovestibular system as well. One of the most severe related consequences is space motion sickness during the first days of spaceflight, but it may also take a toll on higher cognitive functions^3^. Microgravity also has an effect on the sensory-motor system^3^, which is particularly important in tasks that require controlled hand movements, such as tracking and pointing arm movement tasks^4^. When executing a movement, the modified gravity condition can disturb the regular connections among efferent and afferent signals due to altered afferent input from proprioceptors^4^ including the support afferentation system^5^. In addition, other spaceflight-related stressors, such as the elevated CO_2_ level in spacecraft^6^, ionizing radiation^7^, may also impair cognitive functions. The circadian light exposure is also altered in space^3^, which -taken together with other stress factors- may result in sleep problems^8^. Astronauts also have to deal with high workload^3,9^, isolation, and confinement^3^.

**Figure 1:**
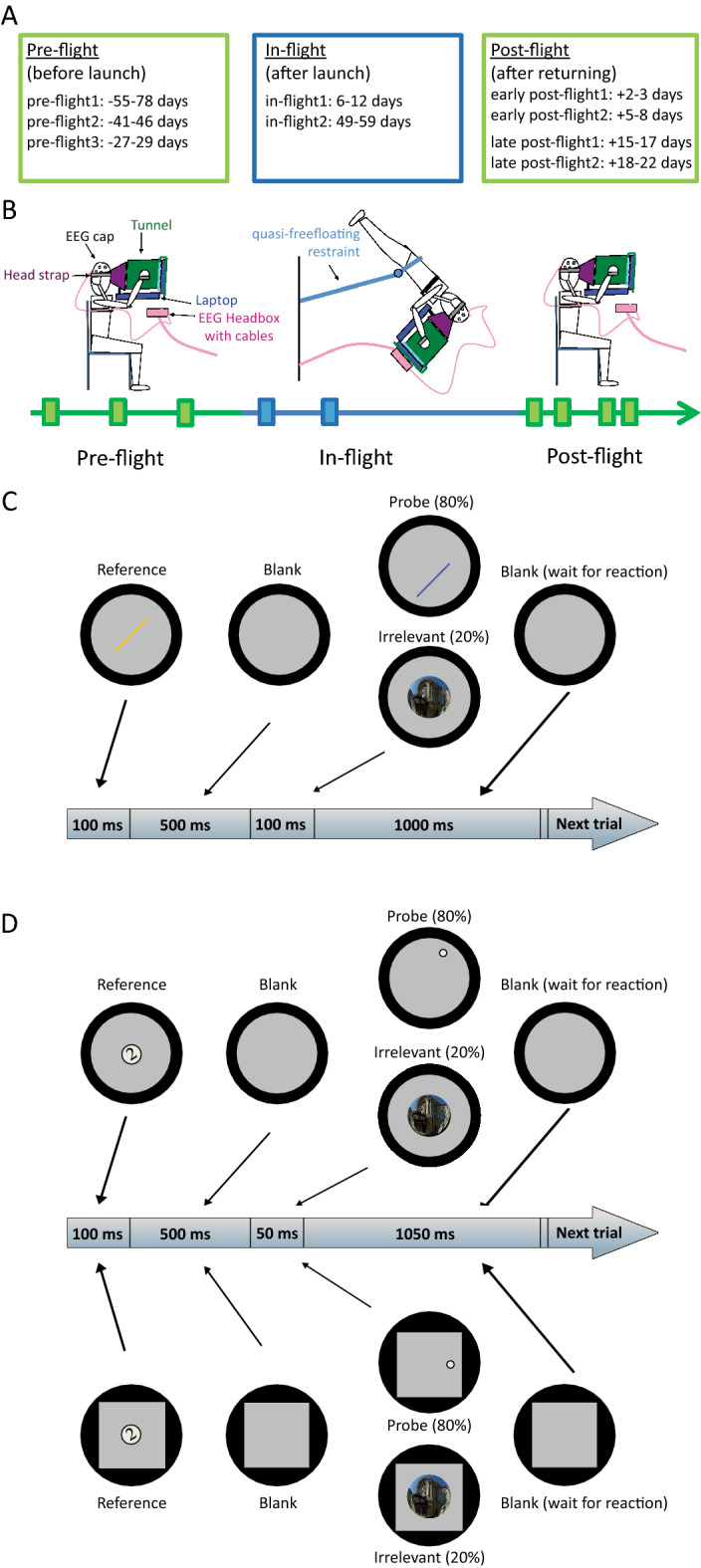
Schematic timeline of study design and the two tasks. (**A**) Schedule of data collection. (**B**) During pre-flight and post-flight, participants performed the experimental tasks in a seated position, watching a computer screen through a facemask attached to a cylindrical tube. On orbit, subjects performed the experiment in a quasi free-floating posture, holding on to the tunnel-computer complex that floated with the subject with the facemask held securely to the head with a strap. (**C**) Stimulus presentation in the Lines task. Participants had to indicate whether two successively presented lines (Reference and Probe stimuli) had the same orientation. 20% of the Probe stimuli were replaced by a task-irrelevant picture (Irrelevant stimulus). (**D**) Stimulus presentation in the Clock task. Participants had to indicate whether the spatial location of a dot (Probe stimulus) matched the clock time represented by a digit (Reference stimulus). Half of the trials were presented in a square frame (Frame condition, shown below), while the other half were presented inside a circular border (NoFrame condition, shown above). The square frame served to provide an oriented visual reference, as the circular tunnel excluded all external visual cues.

The current study aimed to investigate whether prolonged exposure to space environment and related stress factors impact visuospatial functioning during spaceflight. Anecdotal reports by astronauts often mention “space fog”, implying a generalized cognitive slowing at least in the initial period of spaceflight^3^. Although the exact nature of cognitive slowing remains unknown, there are clear indications of altered cognitive processes in a specific domain: visuospatial perception is disturbed in space due to the loss of the key directional cue provided by gravity^10–15^. The visuospatial disturbances are, however, mitigated by increased reliance on preserved sources of reference, such as the head-retinal coordinates as a primary reference, and additionally the visual background information (e.g. orientation of surrounding objects), and body position^16,17^, reflecting the adaptability of the human nervous system. Adaptability may also explain why the majority of studies found no significant deterioration in other cognitive abilities during space travel^9^, and why cognitive problems have never demonstrably posed a serious risk to mission success. However, small sample sizes (or even single case studies^18^), and remarkable variations in timing and frequency of the data acquisition of previous studies limit our understanding of cognition in space. We therefore set out to rigorously investigate cognitive performance in weightlessness by testing astronauts on tasks requiring short-term storage and processing of visuospatial information before (pre-flight), during (in-flight), and after (post-flight) space travel (Fig. 1A,B). Taking into consideration the shortcomings of previous experiments, our study design included multiple data collection points at regular time intervals. By manipulating the amount of available visual cues, we also tested whether humans increase the weight given to visual information in the absence of a stable gravitational reference. Controlled and automatic attentional processes activated during task performance were targeted with late positive event-related potentials (ERP) P3a and P3b^19–21^ of the electroencephalogram (EEG). We hypothesized that spaceflight would negatively affect visuospatial cognitive abilities, which would manifest in deteriorated behavioral performance along with altered event-related brain potentials (P3a and P3b). We also assumed that the absence of gravitational reference would increase reliance on visual information.

## Results

### Impaired visuospatial task performance in space

Two fast-paced, highly taxing visuospatial tasks were applied in our study: the Lines and the Clock task (Fig. 1C,D, and *Methods*). During pre-flight, astronauts performed the two tasks with good general accuracy (Supplementary Figs. S3, S4). Throughout the pre-flight sessions, reaction times showed a strong decreasing trend, reflecting the effect of practice (Supplementary Fig. S1). To avoid biases stemming from this effect, practice effect, estimated from pre-flight data, was subtracted from reaction times (Supplementary Fig. S1).

**Figure 2:**
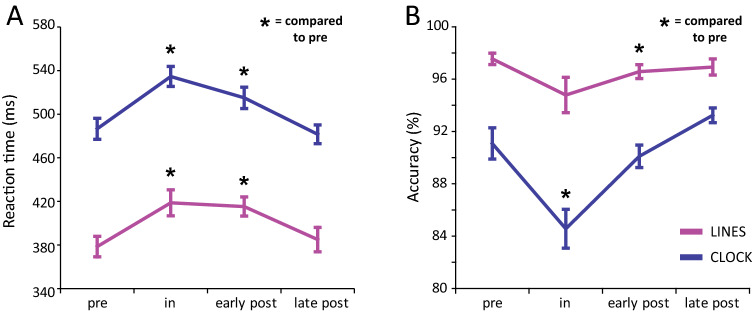
Task performance in the Lines and the Clock task. (**A**) Mean reaction times (corrected for practice effect). Reaction times were significantly slower during space travel compared to pre-flight. Reactions remained slower during early post-flight, but returned to pre-flight levels for late post-flight sessions. (**B**) Mean task accuracy. Accuracy decreased in space in the Clock task. Despite the similar trend, accuracy in the Lines task remained unchanged in space. Accuracy returned to pre-flight levels for late post-flight sessions in both tasks. Error bars represent within-subjects standard error of mean (SEM)^23^. *P < 0.05.

The de-trended reaction times revealed significant microgravity related changes in both tasks (Fig. 2, Supplementary Fig. S2,and Table S2). The two-factor repeated measures ANOVA (rANOVA) [Task (Lines, Clock) × Session (1:9); *Methods*] performed on reaction time, revealed a significant main effect of Session [F(8,32) = 4.06, *P* = 0.03, $\upeta _{{\text{p}}}^{{2}}$ = 0.50]. The Task main effect was also significant [F(1,4) = 35.54, *p* < 0.01, $\upeta _{{\text{p}}}^{{2}}$ = 0.90], with reaction time being longer in the Clock task than in the Lines task. No significant interaction between Task and Session [F(8,32) = 0.79, *P* = 0.51, $\upeta _{{\text{p}}}^{{2}}$ = 0.16] was obtained. The significant Session effect was followed-up by planned contrasts (*Methods*). Contrasts revealed that compared to pre-flight, reaction times were longer during in-flight (*P* = 0.03) and early post-flight (*P* = 0.03), reflecting the negative impact of spaceflight on reaction time. There was a marginal tendency towards further reaction time slowing between the first and the second in-flight measurements (*P* = 0.06). For the late post-flight measurements, reaction time returned back to the pre-flight level (*P* = 0.95).

Accuracy also changed significantly during spaceflight (Fig. 2, Supplementary Figs. S3, S4 and Table S2). A two-factor rANOVA [Task (Lines, Clock) × Session (1:9)] calculated for accuracy revealed a significant main effect of Task [F(1,4) = 9.25, *P* = 0.04, $\upeta _{{\text{p}}}^{{2}}$ = 0.70], Session [F(8,32) = 5.55, *P* = 0.018, $\upeta _{{\text{p}}}^{{2}}$ = 0.58] and interaction between Task and Session factors [F(8,32) = 4.60, *P* = 0.03, $\upeta _{{\text{p}}}^{{2}}$ = 0.53]. Accuracy was lower in the Clock task compared to the Lines task. In order to evaluate the significant interaction between Task and Session, planned contrasts were conducted separately for each task. These tests showed that compared to pre-flight, accuracy in the Clock task was lower during in-flight (*P* = 0.03), but not during early (*P* = 0.52) or late post-flight (*P* = 0.16). As reaction time was increased during in-flight, the drop in accuracy cannot be the result of a simple speed-accuracy tradeoff. The significant decrement of accuracy during in-flight was present for the Clock, but not the Lines task. In the Lines task, only early post-flight differed significantly from pre-flight (*P* = 0.04), with a marginal decrease in-flight (*P* = 0.09) and no difference post-flight (*P* = 0.44). Regarding the two in-flight measurements, there were no significant differences in accuracy in either the Clock (*P* = 0.35), or the Lines task (*P* = 0.16).

### Diminished attentional resources

Changes in controlled and automatic attentional processes were measured with amplitudes of late positive ERP components, P3a and P3b, respectively.

**Figure 3:**
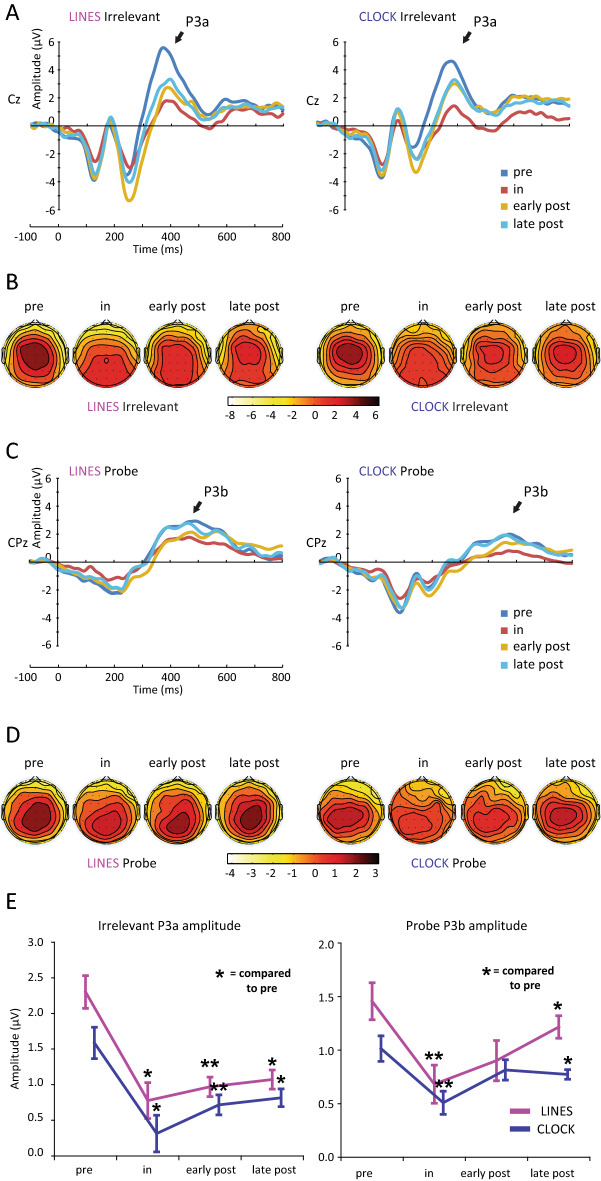
Event-related potentials in the Lines and the Clock task. (**A**) Grand mean ERP elicited by Irrelevant stimuli, presented on the Cz electrode. (**B**) Scalp distribution of P3a component, measured at peak latency (384.8 ms in both tasks). (**C**) Grand mean ERP elicited by Probe stimuli, presented on the CPz electrode. (**D**) Scalp distribution of P3b component, measured at peak latency (468.7 ms for the Lines, and 568.4 for the Clock task). (**E**) Amplitude of P3a and P3b elicited by Irrelevant and Probe stimuli, respectively, averaged over electrodes. P3a and P3b amplitudes showed similar spaceflight related alterations, as both components decreased significantly during in-flight compared to pre-flight. No differences were present between the two in-flight sessions. Compared to pre-flight, amplitudes remained decreased throughout the post-flight period. Error bars represent within-subjects SEM^22^. **p < 0.01; *p < 0.05.

In both tasks P3a was evoked by Irrelevant stimuli. P3a amplitudes decreased considerably in spaceflight. A four-factor rANOVA [Session (1:9) × Task (Lines, Clock) × Region (Frontal, Central, Parietal) × Laterality (Left, Midline, Right)] was calculated for P3a amplitude (Fig. 3, Supplementary Figs. S7, S9, S10A,B). A significant main effect of Task [F(1,4) = 31.02, *p* < 0.01, $\upeta _{{\text{p}}}^{{2}}$ = 0.88] was obtained, with P3a being larger in the Lines compared to the Clock task. The significant Region and Region × Laterality interaction is described in the Supplementary Information. The Session main effect was significant [F(8,32) = 8.49, *p* < 0.01, $\upeta _{{\text{p}}}^{{2}}$ = 0.68]. Planned contrasts showed that compared to pre-flight, P3a amplitude was lower during in-flight (*P* = 0.02), early (*p* < 0.01) and late post-flight (*P* = 0.01). Amplitudes did not differ between the first and the second in-flight measurements (*P* = 0.43) in the Clock or in the Lines task. No other significant main effect or interaction was obtained.

P3b was evoked by Probe stimuli in both the Lines and Clock task. Similarly to P3a, P3b amplitudes also diminished during spaceflight. The four-factor rANOVA [Session (1:9) × Task (Lines, Clock) × Region (Frontal, Central, Centroparietal, Parietal) × Laterality (Left, Midline, Right)] (Fig. 3C,D,E, Supplementary Figs. S8, S9, S10C,D) disclosed a significant Session main effect [F(8,32) = 4.92, *P* = 0.04, $\upeta _{{\text{p}}}^{{2}}$ = 0.55]. The Task main effect was also significant [F(1,4) = 10.54, *P* = 0.03, $\upeta _{{\text{p}}}^{{2}}$ = 0.72]; the amplitude of P3b was larger in the Lines compared to the Clock task. The significant Task × Laterality interaction is detailed in the Supplementary Information. Regarding the Session main effect, planned contrasts revealed that compared to pre-flight, P3b amplitude was lower during in-flight (*p* < 0.01) and late post-flight (*P* = 0.01), with a marginal decrement also in early post-flight (*P* = 0.053). P3b amplitude did not differ significantly between the two in-flight measurements (*P* = 0.49) in the Clock or in the Lines task. No other main effects or interactions were significant. In summary, both P3a and P3b amplitudes showed clear decrements in spaceflight.

### Late onset changes in visual frame effect

To evaluate the effect of visual frame on performance in the Clock task, we also compared reaction time, accuracy and ERP components between the Frame and NoFrame conditions [F(1,4) = 24.93, *p* < 0.01, $\upeta _{{\text{p}}}^{{2}}$ = 0.86]. Subjects were faster in the Frame compared to the NoFrame condition, but no significant differences were found between the two conditions for accuracy, P3a or P3b amplitude, neither were there any cross effects (see Supplementary Information). The lack of interaction between Frame/NoFrame and Session means that, contrary to our hypothesis, we found no convincing evidence that the presence of a visual frame became more salient to the task in weightlessness compared to normal gravity conditions. Nevertheless, a more focused analysis of the visual-frame effect did suggest a gradual shift toward a greater reliance on visual cues over a ~ 50 day exposure to weightlessness (Supplementary Figs. S5, S6, S11, S12, and Table S3).

### Minor decrease in sleep duration and lack of fatigue

Sleep hours varied considerably over the course of the experiment, with large inter-subject variability (Supplementary Fig. S13). Astronauts reported an average of 7.47 h (SD = 0.99) of sleep in preflight, 6.9 h (SD = 0.42) in-flight, 7.8 h (SD = 2.20) in early postflight and 7.4 h (SD = 0.82) in late postflight. Despite the numerical differences, the omnibus ANOVA was not significant [F(8,32) = 0.70, *P* = 0.50, $\upeta _{{\text{p}}}^{{2}}$ = 0.15]. Paralleling the unchanged sleep hours, subjective sleep quality and fatigue (assessed by self-report questionnaires) also remained unaffected (Supplementary Table S4).

## Discussion

Prolonged spaceflight impaired cognitive performance, as evidenced by detectable changes in behavioral performance as well as in ERP. Increases in response times and reduced accuracy were observable during initial (~ 1.5 weeks) and later (~ 1.5–2 months) stages of spaceflight; and may even have deteriorated further over time in weightlessness, as suggested by non-significant trends in the early versus late in-flight comparisons. After returning to Earth, task performance showed a gradual reversal to preflight values, while ERP amplitudes remained diminished over the entire post-flight period, reflecting slow re-adaptation to normal Earth environment.

Our results are therefore in contrast with the majority of previous studies that found no cognitive impairments during spaceflight^9^. For shorter spaceflights, poorer cognitive performance was mainly reported by those who investigated perceptual-motor functions (e.g. tracking and pointing arm movement tasks)^23–25^. With respect to working memory and attentional functions (mainly assessed by dual-tasks and reaction time tasks), a considerable number of studies found intact performance during short-duration space travel^26–28^, while the rest of the studies reported decreased functionality to be either limited to few of the astronauts^29^, or to the initial period of spaceflight^23^. Relatively few studies investigated cognitive functions during prolonged space missions. A portion of these studies found no impairments in weightlessness^16,18,30^, while the rest reported cognitive decrements mainly in perceptual^31^ or perceptual-motor tasks^24,32,33^. Using behavioral measures and event-related potentials, our study revealed impaired performance in tasks requiring working memory and attentional functions during both the initial and later stages of spaceflight.

In line with impaired behavioral performance, a similar pattern of changes was detectable in the investigated ERP components, as both P3b and P3a were reduced during spaceflight. Given that P3b reflects controlled attentional processes^19^; the decrement observed by our study can be related to reduced task accuracy during spaceflight^34–36^. The other component, P3a indexes automatic attentional capacity, commonly associated with the orienting reaction^20,21^. P3a decrement in orbit indicates that participants had reduced capacity to perceive unexpected, novel stimuli. In operational settings, this might mean that during taxing visuospatial tasks astronauts would have less capacity to recognize atypical, possibly threatening events. Additionally, the strong association between performance in visuospatial tasks with task-general executive functions^37^, along with reduced automatic attentional capacity, suggests that the observed poor cognitive performance might not be restricted to visuospatial abilities, but could be a sign of a more general cognitive deterioration.

Several factors could have contributed to the spaceflight-related decrease of cognitive functioning observed by our study. The lack of gravitational reference frame, for example, could play a role in poorer performance. Despite the fact that it is possible to rely on other spatial reference frames (e.g. head-retinal, or visual background) under microgravity^16,18^, it seems that alternative reference systems can not completely substitute for the gravitational one. Indeed, contrary to our predictions, the extra visual frame did not provide much benefit during or after exposure to weightlessness.

As a direct effect of microgravity, the SANS might be implicated in the deterioration of cognitive performance. First, idiopathic intracranial hypertension, a syndrome that presents some of the same neuro-ophthalmological symptoms (chronically elevated intracranial pressure and optic disc swelling) as the SANS, is associated with cognitive symptoms^38,39^. Second, recent studies revealed numerous brain morphology alterations caused by long-duration spaceflight^40–43^ that can be linked to elevated intracranial pressure, a core symptom of SANS. However, there is a factor that argues against the close link between the pathophysiology of SANS and the present case. SANS shows a slow chronic progression, as it is more frequently reported after long, than short-duration spaceflights^43,44^. Also, recovery of changes in brain morphology may not recover even after 6 months after return^45^. Although in our study a relatively fast-paced time course of changes was observable (diminished performance during the first in-flight and improved performance during late post-flight sessions). The spaceflight environment has also been suggested to precipitate slower cerebral circulation due to impaired venous drainage^46^, however, previous cerebral blood flow measurements in space and in simulated microgravity using Doppler sonography^47–57^ and phase-contrast MRI^58,59^ reported conflicting results. Future studies should investigate cerebral circulation, ophthalmic and structural brain changes jointly with cognitive assessment. As for space motion sickness, the time course of symptom resolution is estimated between 12 to 72 h^13^, which makes it unlikely to affect in-flight measures (conducted 6–12, and 49–59 days after the launch). Conversely, space motion sickness could have contributed to the poor performance observed at the first early post-flight measure, which was conducted shortly (2–3 days) after returning to Earth. Concerning the effect of microgravity on the sensory-motor system, the influence on the present finding on reaction times cannot be excluded, however, our tasks are not typically perceptual-motor tasks like tracking or pointing arm movement. Nevertheless, motor slowing cannot explain the deterioration of accuracy.

Aside from weightlessness, other spaceflight-related factors could also contribute to the observed cognitive deficits. For instance, elevated CO_2_ concentrations inside the International Space Station (ISS) can be a significant stressor^7^. Although a chronic (26-day long) terrestrial simulation reported only minor decrements in visuomotor performance, while no functions requiring working memory and cognition were affected^60^. Due to the protective effect of Earth's magnetic field, space radiation is assumed to be less of a concern for astronauts living on low Earth orbit (i.e. ISS)^8^. Another spaceflight-related factor, the high workload experienced by astronauts can lead to mental fatigue and damaged attention. Several space studies found deterioration in performance along with increased subjective fatigue and/or workload [e.g.^3,25,27^. Furthermore, a study^61^ showed that P3a could be sensitive to mental fatigue. Therefore, high workload could have contributed to our findings. As for isolation, and confinement on the ISS, there is no clear evidence whether it negatively affects cognitive functions. Present results are in good accordance with the previous findings that astronauts show a small, but clear (~ 30 min) decrease in sleep duration onboard the ISS^9^. As ground-based studies are lacking regarding the significance of such amounts of sleep loss over an extended period of time, the adverse effect of reduced sleep duration cannot be excluded.

Our pilot study is the first to provide evidence for impaired visuospatial performance during both the initial (~ 8 days) and later (~ 50 days) stages of spaceflight. However, some limitations regarding our study design should be noted. First, while small sample size is quite common in space-related research due to practical reasons, the number of subjects in our study is low, therefore, our study should be considered as a pilot study. Second, the lack of ground-control group is also a shortcoming of our design, however we believe that the number of baseline sessions provide a firm basis to distinguish space related changes. Additionally, in-flight sessions were performed in a quasi free-floating position while baseline measurements were performed in a seated position, nonetheless, the gradual return of the observed differences after landing argues against the causal role of body position. Besides that, our results are limited to the visual domain, which calls for additional research to extend the findings to other modalities and cognitive domains.

Nevertheless, our finding of decreased visuospatial cognitive performance and attentional resources during a prolonged stay on the ISS extends the knowledge of possible challenges related to the future exploration of our solar system and beyond, where astronauts are exposed to the above mentioned factors for a longer period of time. At greater distances from Earth, mission success will depend even more on crewmembers’ abilities to perform complex visuospatial maneuvers and to quickly react to anomalous situations aboard their spacecraft.

## Methods

### Subjects

Five male astronauts with a mean age of 54.2 (SD = 2.6) participated in this experiment. Each astronaut took part in an approximately half-year long space mission aboard the ISS. This study was conducted during four missions (expeditions 20/21, 26/27, 30/31, 34/35). Tasks were performed on 9 occasions, 3 times before, 2 times during and 4 times after flight. Data were collected at regular time intervals (Fig. 1A). A detailed testing schedule is also available here^62^. Pre-flight and post-flight data recordings were conducted at the Gagarin Cosmonaut Training Center (Star City, Russia), at the European Astronaut Centre (Cologne, Germany), and at the Johnson Space Center (Houston, USA). All of the astronauts had previously participated in short space missions.

The Ethics Committee of the Institute of Cognitive Neuroscience and Psychology, Research Centre for Natural Sciences (formerly known as the Institute for Psychology of the Hungarian Academy of Sciences), the Institutional Review Board (IRB) of the European Space Agency (ESA), and the IRB of NASA approved the experimental protocol, which complied with all guidelines stated in the Declaration of Helsinki. All astronauts signed an informed consent form on the experimental procedures.

### Stimuli and procedure

In order to minimize fatigue, tasks were presented in 5–8 min blocks with short breaks in-between. Blocks of tasks were arranged in a way to minimize differences due to practice/fatigue building up in the course of a session. The order of task blocks was as follows: Control measurements, one block of Lines task, one block of Clock task NoFrame condition, one block of Clock task Frame condition, four blocks of Visuomotor Tracking task, one block of Clock Frame task condition, one block of Clock task NoFrame condition, and one block of Lines task. The total execution time was 70 min for each session. This paper is focused on the results of the Lines and the Clock tasks, for results regarding the Visuomotor Tracking task, see^62^. The Lines and the Clock tasks were both developed for this study to assess the influence of weightlessness on the perception of spatial directions as well as attention-related ERP components.

Subjects were equipped with an EEG cap and looked straight ahead at a laptop screen through a form-fitting facemask attached to a cylindrical tunnel. The tunnel excluded external visual cues and provided a circular viewing field. The screen was centered on the line of gaze at a distance of 25 cm from the eyes. The tunnel had a diameter of 22 cm. The background color of the screen was dark gray. During pre-flight and post-flight sessions, participants performed the experimental tasks in a seated position in a quiet room at a computer desk, while focusing on a computer screen through a facemask attached to a cylindrical tube. During flight, subjects performed the experiment in a quasi free-floating posture in the Columbus module of the ISS, while holding on to the tunnel-computer complex with the facemask strapped to the forehead with an adjustable belt. They had no rigid contact with the station structure during the performance of the experiment (Fig. 1B).

Prior to the first session, subjects were familiarized with the procedure and practiced the experimental tasks during two sessions separated by at least six days. Subjects were trained to perform the in-flight measurements themselves with the help of a fellow crewmember.

### Lines task

First, a simple yellow line appeared on the screen for 100 ms, presented on a light gray disk (with a diameter of 16.8 cm) (Fig. 1C). The orientation of this *Reference* stimulus had to be remembered. Following a 500 ms blank screen, a *Probe* stimulus (a blue line) was presented for 100 ms. The orientation of the Reference and the Probe stimuli could either be identical or could differ by 30 or 60 degrees with equal probability. Subjects had to indicate whether the Probe stimulus had the same orientation as the Reference stimulus by pressing a pushbutton on a gamepad as quickly as possible. If the lines had the same orientation, a button had to be pressed with the right index finger, while another button had to be pressed with the right thumb if the lines had different orientations. Subjects had 900 ms to react after Probe stimulus onset. The time between consecutive trials (stimulus onset asynchrony, SOA) was 1700 ms. In 20% of the trials, a task-irrelevant stimulus (a picture) was presented for 100 ms instead of the Probe stimulus. Subjects were instructed not to press any button in response to *Irrelevant* stimuli. Reference and Probe lines were both 2.1 cm in length and 0.2 cm in width. Irrelevant stimuli were various colorful, circular, fisheye pictures with a diameter of 4.7 cm showing buildings, statues, and everyday objects. The Lines task consisted of 450 trials divided into 2 blocks. In the first 10 trials of the first block, an auditory feedback (beep) was provided when the subject responded erroneously.

### Clock task

At the beginning of each trial, a black number (*Reference* stimulus) representing clock time was presented on a light gray disk (representing the face of a digital clock) with a diameter of 1.1 cm for 100 ms (Fig. 1D). The following numbers were used as Reference stimuli: 1, 2, 4, 5, 7, 8, 10 and 11. Numbers were rotated randomly by ± 60º, ± 30º, or 0º. Following a 500 ms blank screen, a white *dot* (*Probe* stimulus) was displayed on the perimeter of an invisible circle (representing the face of an imaginary analog clock) for 50 ms. The diameter of the dot was 0.4 cm. Subjects had to indicate as quickly as possible whether the location of the dot corresponded to the digital clock time indicated by the Reference stimulus. By using a gamepad, subjects were instructed to press a button with the right index finger if the location of the dot matched the clock time and to push another button with the right thumb if the dot did not match the clock time. Subjects had 900 ms to react after the Probe stimulus onset. SOA was 1700 ms. The Probe stimulus could either match the clock time indicated by the Reference stimulus or could differ from it by 1 or 2 h with equal probability. As in the Lines task, task-irrelevant stimuli were presented instead of the Probe stimulus in 20% of the trials. Irrelevant stimuli were various fisheye pictures in response to which subjects were instructed not to press any button.

The Clock task consisted of 600 trials divided into 4 blocks. In order to foster spatial orientation, a squared frame was presented around the visible workspace on the screen (Frame condition; with a 10.5 cm × 10.5 cm frame) in one half of the blocks. In the other half of the blocks, instead of a squared frame, stimuli were presented in a circle (NoFrame condition) with a diameter of 12.1 cm. In the first 10 trials of the first block of both conditions, auditory feedback (a beep) was provided when the subject pushed the wrong key.

### EEG recording and analysis

In all in-flight and pre-flight measurements as well as in 30% percent of the post-flight measurements, EEG was recorded with the Multi-Electrodes Encephalogram Measurement Module (MEEMM, specifically created for the ISS by OHB Systems, Germany), with a sampling frequency of 1116 Hz. 58 scalp electrodes were placed according to the extended 10–20 system; the ground electrode was placed on the forehead. In the other 70% of the post-flight measurements, EEG was recorded with the ANT system (ANT Neuro, The Netherlands) with a sampling frequency of 1024 Hz. 59 scalp electrodes were placed using the extended 10–20 system while an additional scalp electrode (AFz) functioned as ground. It is worth noting that only comparisons with post-flight measurements could have been influenced by the different EEG systems, however, the two setups were compared (same day, same subjects) and no observable differences were found.

Horizontal eye movements were monitored using two electrodes placed lateral to the outer canthi of each eye and vertical eye-movements were monitored with an electrode placed below the left eye. A right-ear reference was used for all recordings. EEG was analyzed with the EEGLAB toolbox^63^. EEG was bandpass filtered offline (0.5–40 Hz, Kaiser windowed sinc FIR filter) and down-sampled to 512 Hz. Large, noisy time segments and channels were removed after visual inspection. Extended independent component analysis (ICA) was performed on individual data sets to remove eye blink artifacts from EEG recordings^63,64^. ICA components representing eye blink and horizontal eye movement artifacts were identified by inspecting the component scalp maps, time courses and ERP-images (visualization of event-related signal variations across single trials) and were deleted. Missing data (channels) were interpolated using spherical spline interpolation. Processed EEG was re-referenced to the average of the signal of all electrodes and was lowpass filtered at 30 Hz (Kaiser windowed sinc FIR filter).

1000 ms-wide epochs were extracted (100 ms pre-probe to 900 ms post-probe) and baseline corrected (100 to 0 ms pre-probe) in each task. Only trials with correct responses were analyzed. Epochs with a signal range exceeding 70 µV on frontal, central, and temporal channels and 100 µV on parietal and occipital channels (where alpha oscillations frequently exceed 70 µV) were discarded from the analyses. Grand-means were computed from individual averages.

### Data analysis

To eliminate reactions reflecting fast guesses, only correct responses with a duration greater than 200 ms after Probe offset were included in the analyses of reaction time. Median reaction time was calculated for each session, stimulus type, and subject in both tasks.

Accuracy was calculated as the percentage of correct button presses for Probe stimuli in both tasks. To assess the effect of spaceflight on both reaction time and accuracy, two-factor [Task (Lines, Clock) × Session (1:9)] rANOVAs were conducted. In order to evaluate the effect of visual frame in the Clock task, separate two-factor [Visual Frame (Frame, NoFrame) × Session (1:9)] rANOVAs were performed on both reaction time and accuracy.

The ERP analysis focused on the P3a ERP component elicited by Irrelevant stimuli and the P3b component elicited by Probe stimuli. As the P3a component elicited by Lines and Clock Irrelevant stimuli showed similar waveforms and peak latencies, P3a peak latency was determined on the overall mean ERP waveforms averaged across subjects, sessions, and tasks at the Cz electrode site within the range of 280–480 ms after the onset of Irrelevant stimuli (collapsed localizer method^65^). P3a amplitude was evaluated in a 100 ms wide time window centered at averaged peak latency. Mean amplitude values were analyzed using Session (1:9) × Task (Lines, Clock) × Region (Frontal: F3, Fz, F4; Central: C3, Cz, C4; Parietal: P3, Pz, P4) × Laterality (Left: F3, C3, P3; Midline: Fz, Cz, Pz; Right: F4, C4, P4) rANOVA. To assess the effect of visual frame in the Clock task, a separate rANOVA was conducted with Session (1:9) × Visual Frame (Frame, NoFrame) × Region (Frontal: F3, Fz, F4, Central: C3, Cz, C4, Parietal: P3, Pz, P4) × Laterality (Left: F3, C3, P3, Midline: Fz, Cz, Pz, Right: F4, C4, P4) as independent factors.

P3b peak latencies and peak amplitudes were computed for Probe stimuli. Since P3b latency was significantly different between the Lines and the Clock task, latency ranges for mean amplitude measurements were defined separately for the two tasks. Using the collapsed localizer method, P3b peak latencies were identified in the grand-mean ERP waveforms averaged across subjects and sessions for the Lines and Clock tasks separately at the CPz electrode site within 350–700 ms after the onset of Probe stimuli. P3b peak latency was 468.7 ms for the Lines, and 568.4 ms for the Clock task. P3b amplitude was evaluated in a 100 ms time window centered at peak latency. Mean amplitude values were analyzed using a Session (1:9) × Task (Lines, Clock) × Region (Frontal: F3, Fz, F4; Central: C3, Cz, C4; Centroparietal: CP3, CPz, CP4; Parietal: P3, Pz, P4) × Laterality (Left: F3, C3, CP3, P3; Midline: Fz, Cz, CPz, Pz; Right: F4, C4, CP4, P4) rANOVA. The effect of visual frame in the Clock task was evaluated using Session (1:9) × Visual Frame (Frame, NoFrame) × Region (Frontal: F3, Fz, F4; Central: C3, Cz, C4; Centroparietal: CP3, CPz, CP4; Parietal: P3, Pz, P4) × Laterality (Left: F3, C3, CP3, P3; Midline: Fz, Cz, CPz, Pz; Right: F4, C4, CP4, P4) rANOVA.

Greenhouse–Geisser correction was applied for all repeated measures with greater than 1 degree of freedom. Uncorrected degrees of freedom and corrected *p* values are reported. Partial eta squared ($\upeta _{{\text{p}}}^{{2}}$) was computed as an estimate of effect size.

In case the omnibus ANOVA showed significant Session main or cross effects, planned contrasts were performed to compare sessions grouped into in-flight, early and late post-flight with the pre-flight baseline (see Fig. 1A). The difference between the two in-flight measurements was also investigated with contrasts. The same set of contrasts was applied on all outcome variables. Any effects involving the Region or Laterality factors in the ERP analysis were treated as post-hoc effects and were specified by Tukey-HSD tests. Given that tests of normality have low power with small sample size, all a priori contrasts were checked with a non-parametric alternative (Friedman test), which lead to parallel results in all cases. Individual data are also depicted in the Supplementary Information (Supplementary Figs. S4, S9, S10).

## Supplementary Information

Supplementary Information

## Data Availability

Data analysis scripts and data underlying the statistical analysis are available on figshare (https://figshare.com/s/65feabaa1e69683f8cad).
