# Supplementary material for: Persistent deterioration of visuospatial performance in spaceflight
Source: Sci Rep. 2021 May 5;11:9590. doi: 10.1038/s41598-021-88938-6 (PMC8100295; doi:10.1038/s41598-021-88938-6)
Supplement: Supplementary file 1 — Supplementary Information [file 41598_2021_88938_MOESM1_ESM.pdf]

Supplementary information

Persistent deterioration of visuospatial performance in spaceflight

Endre Takács, Irén Barkaszi, István Czigler, Lívia Gabriella Pató, Anna Altbäcker, Joseph McIntyre, Guy Cheron, László Balázs

Methods

Practice effect

Reaction times showed a strong decreasing trend throughout pre-flight sessions, reflecting an effect of repetition or practice (Supplementary Fig. S1). As comparing in-flight reaction times to the average of pre-flight values might be misleading, the practice effect was modeled based on pre-flight measurements (session 1, 2 and 3), as follows.

Following theoretical considerations [1], a power function was chosen for modeling practice effect, which was fit to pre-flight data averaged across subjects and then subtracted from individual datasets across all sessions (pre-, in- and post-flight). The equation of the power function was $Y = a\, X^{m}$, where Y represents reaction time, X stands for session number, while *a* and *m* reflect the rate and acceleration (deceleration) of the learning effect. In order to estimate these parameters, a linearization of the power function was performed by applying a log/log transformation. The R^2 value of power-law curve fitting was 0.97 for the Lines task, and 0.68 for the Clock task. After the estimation of *a* and *m*, the practice effect was subtracted from the individual Y values, i.e. $Y' = Y - a\,(X^{m} - 5^{m})$, where 5 is the midpoint of the 1-9 measurement points.

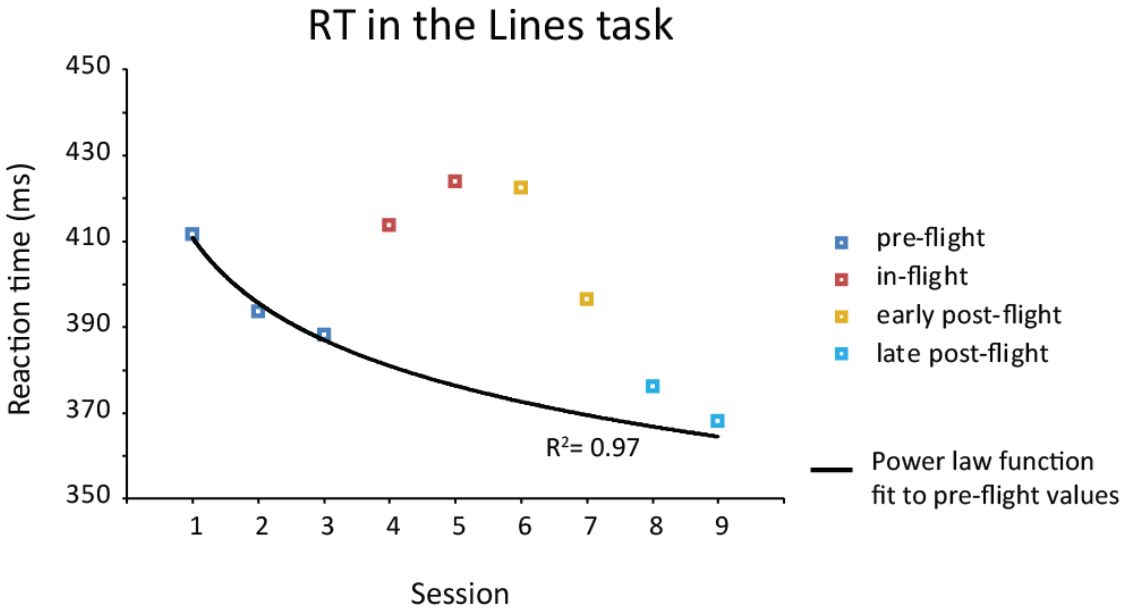

Supplementary Figure S1. Mean reaction times of the Lines Task before correction for practice effect.

Visual frame effect

For the visual frame effect, we applied planned contrasts regardless of the outcome of the corresponding ANOVA. Due to the rarity of these kinds of data, and because of our strong a priori hypothesis about a

potential effect of gravity on the visual frame effect based on other studies, we feel that reporting these results is informative and warranted to guard against a type II statistical error.

Sleep and fatigue

Answers to questions regarding subjective sleep quality, fatigue and sleepiness were recoded to numerical values (Supplementary Table S1). Due to lack of nonparametric alternatives of planned contrasts, these variables were analyzed solely by one-factor [Session (1:9)] Friedman ANOVAs.

Supplementary Table S1. Questions regarding subjective sleepiness and fatigue.

**question**	**short name**	**response alternatives**			
How many times did you wake up during the night?	number of awakenings	once (0)	twice (1)	3-4 times (2)	5 times or more (3)
Did you feel excessively sleepy in the morning after awakening?	morning sleepiness	no (0)	yes (1)		
Did you feel excessively sleepy during the day?	daytime sleepiness	no (0)	yes (1)		
How Fit/Tired did you feel today?	daytime fatigue	very fit (0)	not fit, but not tired (1)	more tired than usual (2)	very tired (3)
How would you rate your sleep?	sleep quality	much better than usual (0)	as usual (1)	worse than usual (2)	much worse than usual (3)
When did you fall asleep? (approximately)<br>When did you wake up? (approximately)	sleep hours				

Responses were recoded to numerical values, shown in parentheses.

Results

Task performance

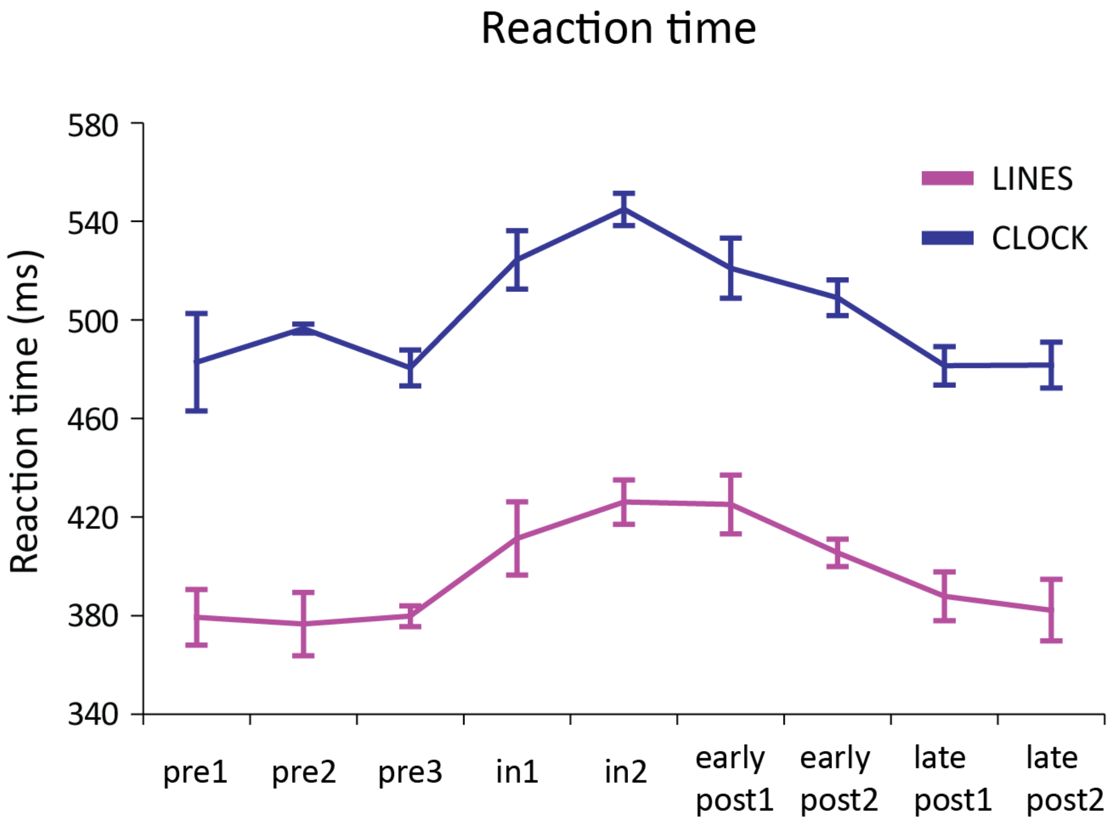

Supplementary Figure S2. Mean reaction time in the Lines and Clock task over all 9 sessions, corrected for practice effect. Error bars denote within-subjects standard error of mean (SEM) [2].

Supplementary Table S2. Results of the planned contrasts between flight conditions.

		**in-flight vs. pre-flight**			**in-flight1 vs. in-flight2**			**early post-flight vs. pre-flight**			**late post-flight vs. pre-flight**		
		F	p	η_p^2	F	p	η_p^2	F	p	η_p^2	F	p	η_p^2
RT		10.03	0.03*	0.91	6.65	0.06	0.62	10.77	0.03*	0.73	<0.01	0.95	<0.01
AC	Li	5.04	0.09	0.56	2.99	0.16	0.43	8.74	0.04*	0.69	0.74	0.44	0.16
	Cl	11.07	0.03*	0.73	1.12	0.35	0.22	0.50	0.52	0.11	2.91	0.16	0.42
P3a		14.66	0.02*	0.78	0.76	0.43	0.16	30.87	0.005*	0.89	17.35	0.01*	0.81
P3b		23.50	0.008*	0.85	0.56	0.49	0.12	7.40	0.053	0.65	19.77	0.01*	0.83

RT: Reaction time; AC: Accuracy; Li: Lines task, Cl: Clock task; *P < 0.05; **P < 0.01

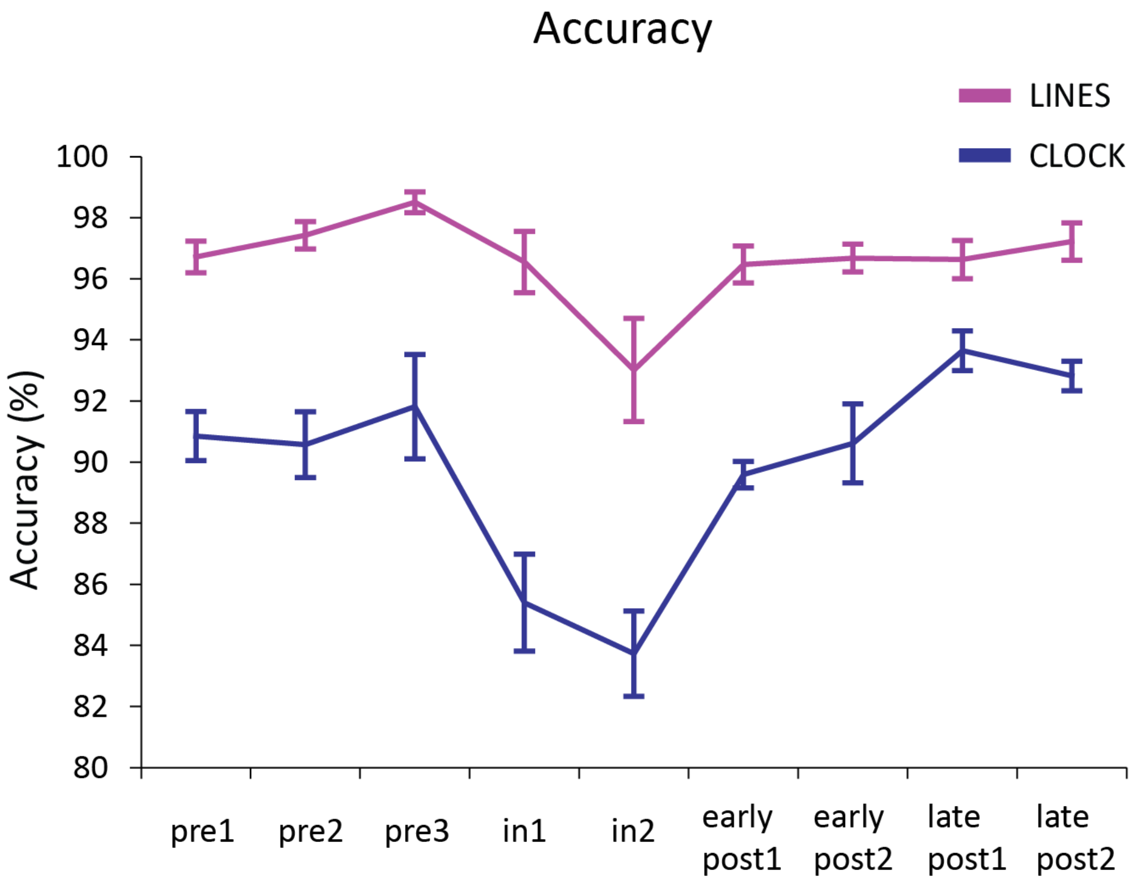

Supplementary Figure S3. Mean accuracy in the Lines and Clock task over all 9 sessions. Error bars denote within-subjects SEM [2].

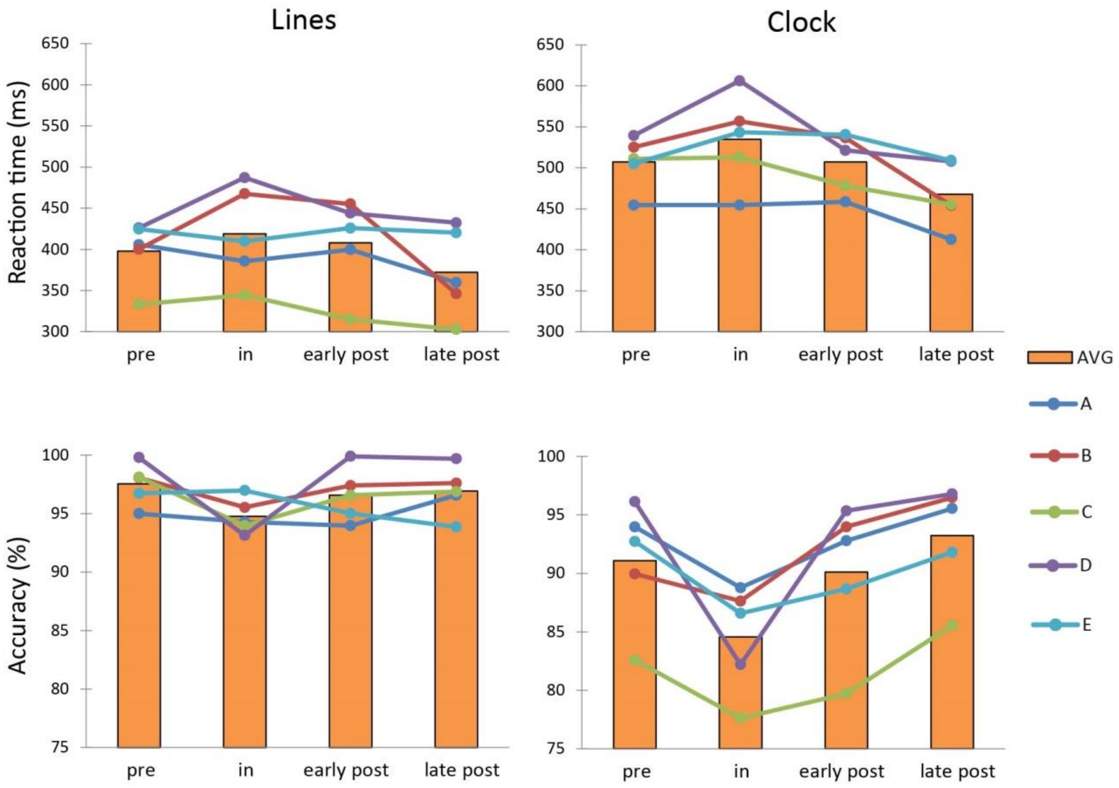

Supplementary Figure S4. Mean (AVG) and individual (A, B, C, D and E subjects) task performance in the Lines (left) and the Clock (right) tasks over pre-, in-, early post- and late post-flight; uncorrected Reaction Time (upper panels) and Accuracy (bottom panels)

Visual frame effect on task performance

In order to evaluate the visual frame effect in the Clock task, two-factor ANOVAs [Visual Frame (Frame, NoFrame) × Session (1:9)] were performed on reaction time (Supplementary Fig. S5) and accuracy (Supplementary Fig. S6). A significant main effect of Visual Frame on reaction time [$F(1,4) = 24.93$, $P < 0.01$, $\eta_p^2 = 0.86$] but not on accuracy [$F(1,4) = 4.25$, $P = 0.11$, $\eta_p^2 = 0.51$] shows that reaction time was longer in the NoFrame condition than in the Frame condition along with essentially the same level of accuracy. Although no significant interactions between Visual Frame and Session were found in either case [reaction time: $F(8,32) = 1.49$, $P = 0.20$, $\eta_p^2 = 0.27$; accuracy: $F(8,32) = 2.48$, $P = 0.11$, $\eta_p^2 = 0.38$], planned contrasts did reveal a notable increase of the frame effect for accuracy in the second in-flight session, compared to the first [$F(1,4) = 29.62$, $P < 0.01$, $\eta_p^2=0.88$] (Supplementary Fig. S6). Indeed, while accuracy for Frame and NoFrame appears to be equal for the preflight and late post-flight sessions, the increased divergence between Frame and NoFrame for the second in-flight session shows the aforementioned increased frame effect. In addition, while not statistically significant compared to the pre-flight period [$F(1,4) = 5.33$, $P = 0.08$, $\eta_p2 = 0.57$], the incomplete convergence of Frame and NoFrame for the first and second early post-flight sessions, suggests the possibility of a gradual readjustment to normal gravity conditions. The combined in-flight accuracy also showed significant difference with respect to the combined pre-flight level [$F(1,4) = 9.37$, $P = 0.04$, $\eta_p^2 = 0.70$], however this effect is attributable to the second in-flight session, as accuracy was largely equivalent between Frame and NoFrame conditions in the first in-flight session. No concomitant effect of Session on the frame effect was found for reaction time (for planned contrasts, see Supplementary Table S3), indicating that the observed effect for accuracy

was not simply the result of a more hurried performance of the experiment during the second in-flight session. These observations argue against an immediate increased reliance on visual reference frames during spaceflight, in contrast to our initial hypothesis, but suggest a gradual shift toward greater reliance on visual cues over ~50 days exposure to weightless conditions.

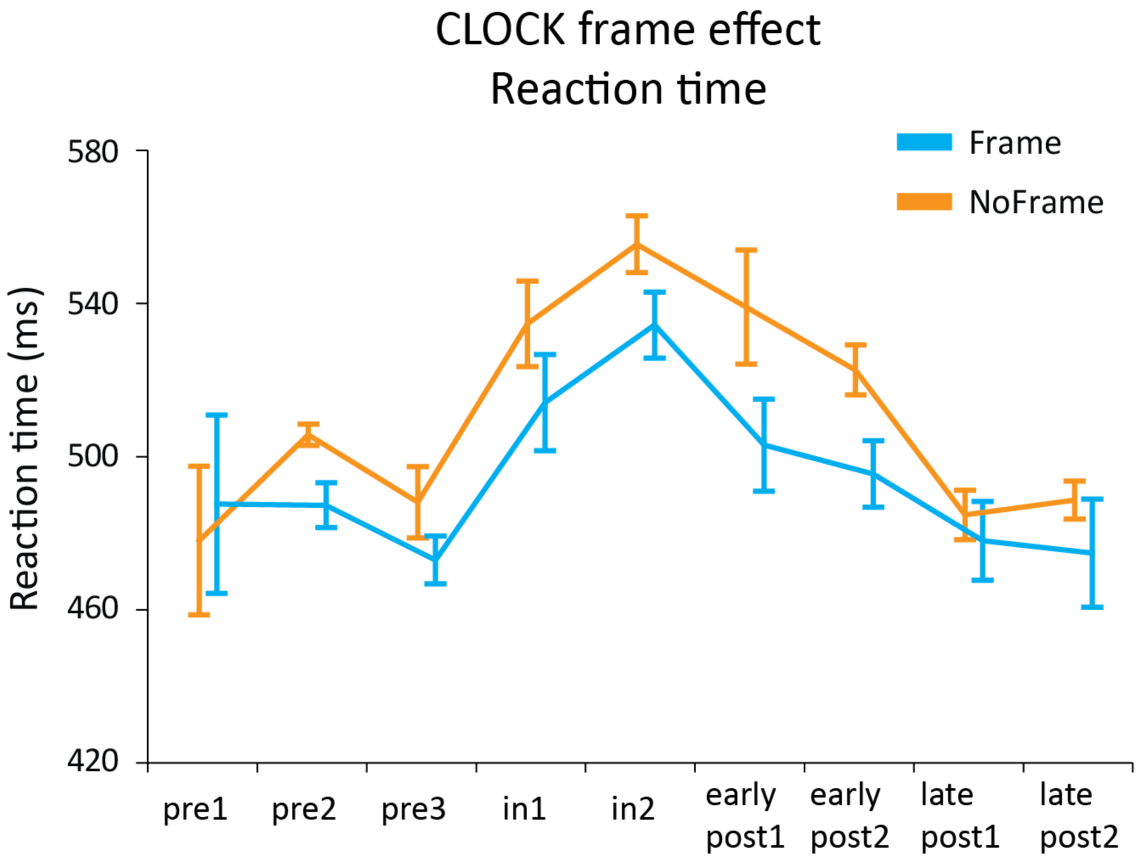

Supplementary Figure S5**.** Mean reaction time in Frame and NoFrame conditions of the Clock task in over all 9 sessions. Corrected for practice effect. Error bars denote within-subjects SEM [2].

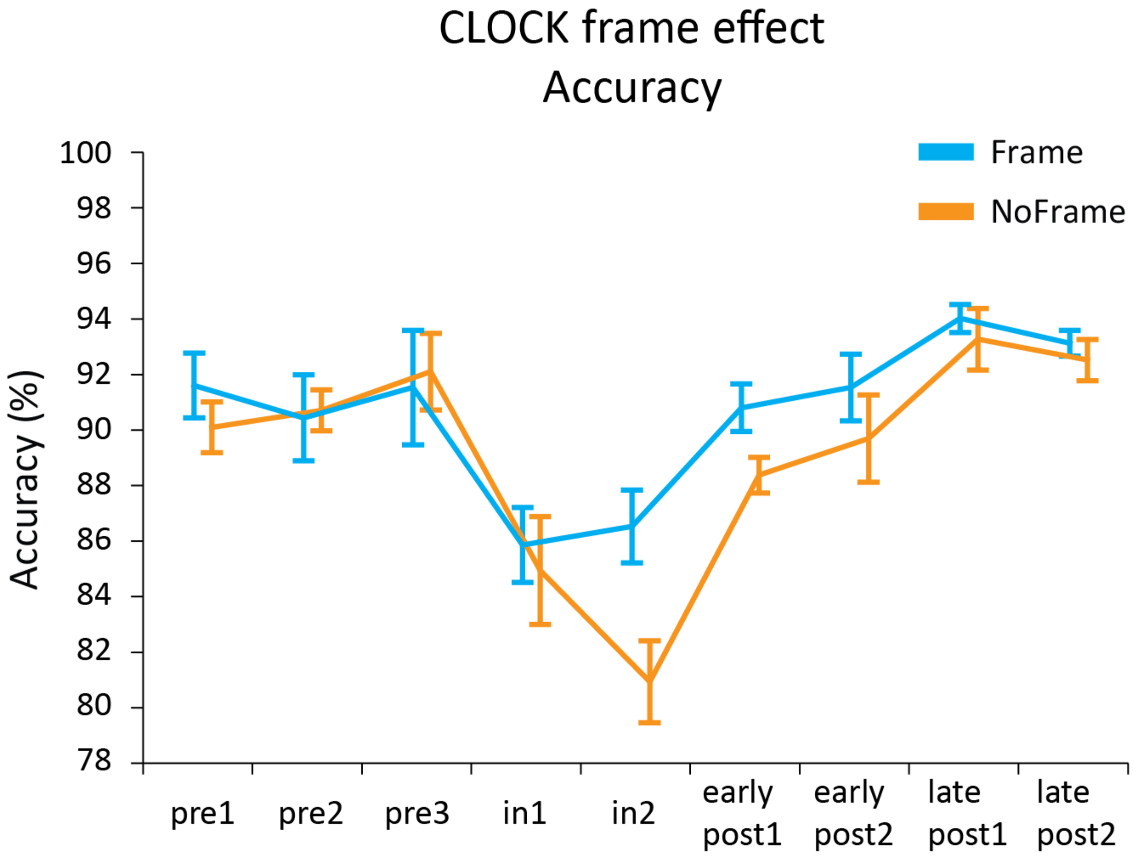

Supplementary Figure S6. Mean accuracy in Frame and NoFrame conditions of the Clock task in over all 9 sessions. Error bars denote within-subjects SEM [2].

Supplementary Table S3. Results of the planned contrasts between flight conditions for the visual frame effect (Clock task).

	**in-flight vs. pre-flight**			**in-flight1 vs. inflight2**			**early post-flight vs. pre-flight**			**late post-flight vs. pre-flight**		
	F	P	η_p^2	F	P	η_p^2	F	P	η_p^2	F	P	η_p^2
RT	1.29	0.32	0.24	<0.01	0.96	<0.01	5.12	0.09	0.56	0.03	0.87	<0.01
AC	9.37	0.04*	0.70	29.62	0.006**	0.88	5.33	0.08	0.57	0.28	0.63	0.06
P3a	1.80	0.25	0.31	0.28	0.62	0.07	0.35	0.59	0.08	1.72	0.26	0.30
P3b	0.51	0.51	0.11	1.78	0.25	0.31	0.04	0.85	<0.01	0.13	0.73	0.03

RT=reaction time; AC=Accuracy; *P < 0.05; **P < 0.01

Event related potentials

P3a elicited by Irrelevant stimuli

A four-factor repeated measures ANOVA (rANOVA) [Session (1:9) × Task (Lines, Clock) × Region (Frontal, Central, Parietal) × Laterality (Left, Midline, Right)] was calculated for P3a amplitude. In addition to the effects presented in the article, a significant main effect of Region [$F(2,8) = 7.63$, $P = 0.03$, $\eta_p^2 = 0.66$] and a significant Region × Laterality interaction [$F(4,16) = 4.58$, $P = 0.03$, $\eta_p^2 = 0.53$] were also obtained. P3a amplitude was smaller at frontal electrode sites compared to central ($P = 0.02$) and parietal regions ($P = 0.03$). The Region × Laterality interaction was driven by the different laterality effects in each cortical region: while in the central region amplitudes were larger in the midline compared to both lateral sites (all P values < 0.01), in the frontal region only the right site differed from the midline site ($P = 0.01$; left vs. midline site $P = 0.96$), while no laterality effect emerged in the parietal region (all P values > 0.20).

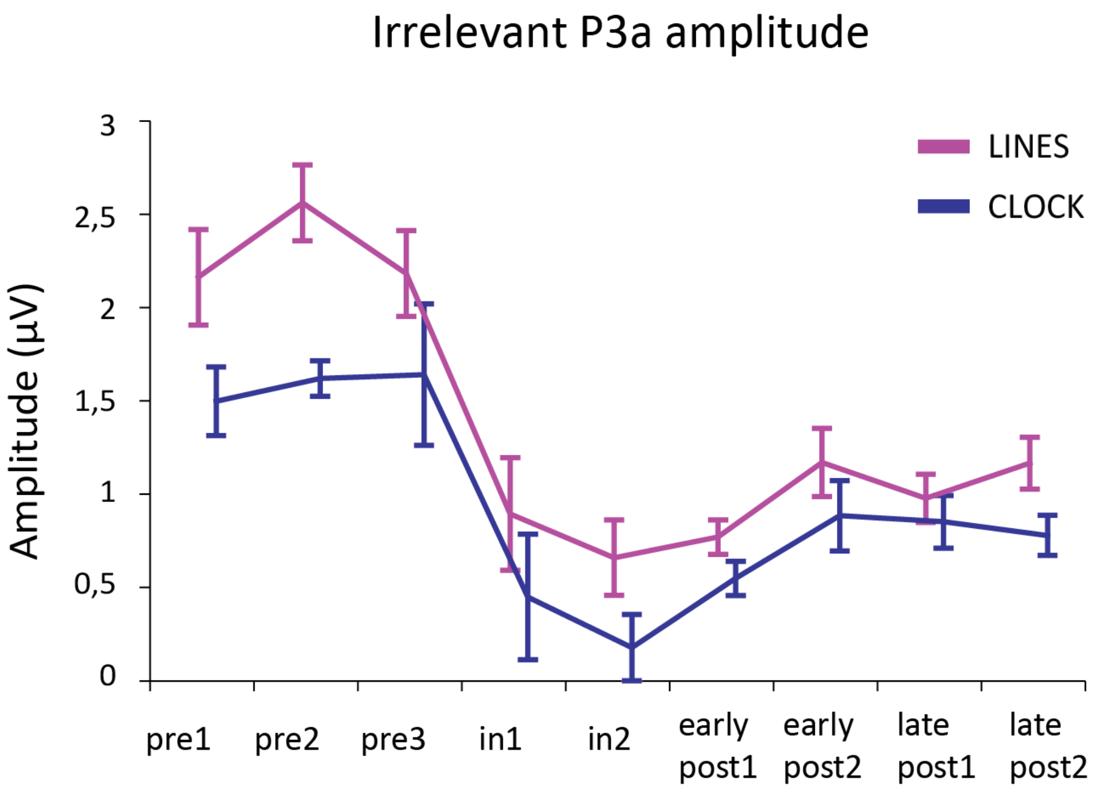

Supplementary Figure S7. Mean amplitude of P3a elicited by Irrelevant stimuli in both tasks over all 9 sessions. Amplitudes are averaged over the analyzed electrode matrix. Error bars denote within-subjects SEM [2].

P3b elicited by Probe stimuli

In addition to the significant effects described in the article, a significant Task × Laterality interaction [$F(2,8)=25.68$, $p<0.001$, $\eta_p^2=0.86$] was also obtained. In general, as the significant Task main effect supports, the amplitude of P3b was larger in the Lines compared to the Clock task. The Task × Laterality interaction reflects the modulatory effect of Laterality on the Task effect: P3b amplitudes were only larger in the Lines compared to Clock task at midline ($P < 0.001$) and right sites ($P < 0.001$), but not at left sites ($P = 0.99$).

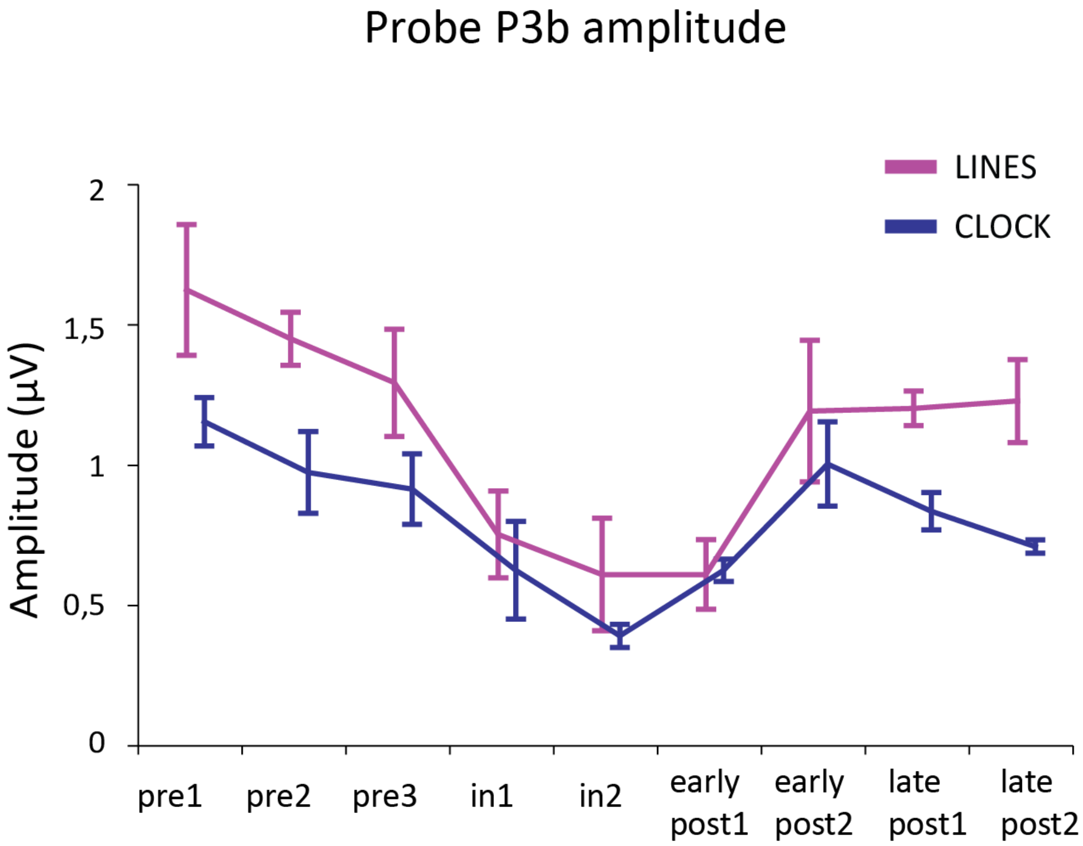

Supplementary Figure S8. Mean amplitude of P3b elicited by Probe stimuli in both tasks over all 9 sessions. Amplitudes are averaged over the analyzed electrode matrix. Error bars denote within-subjects SEM [2].

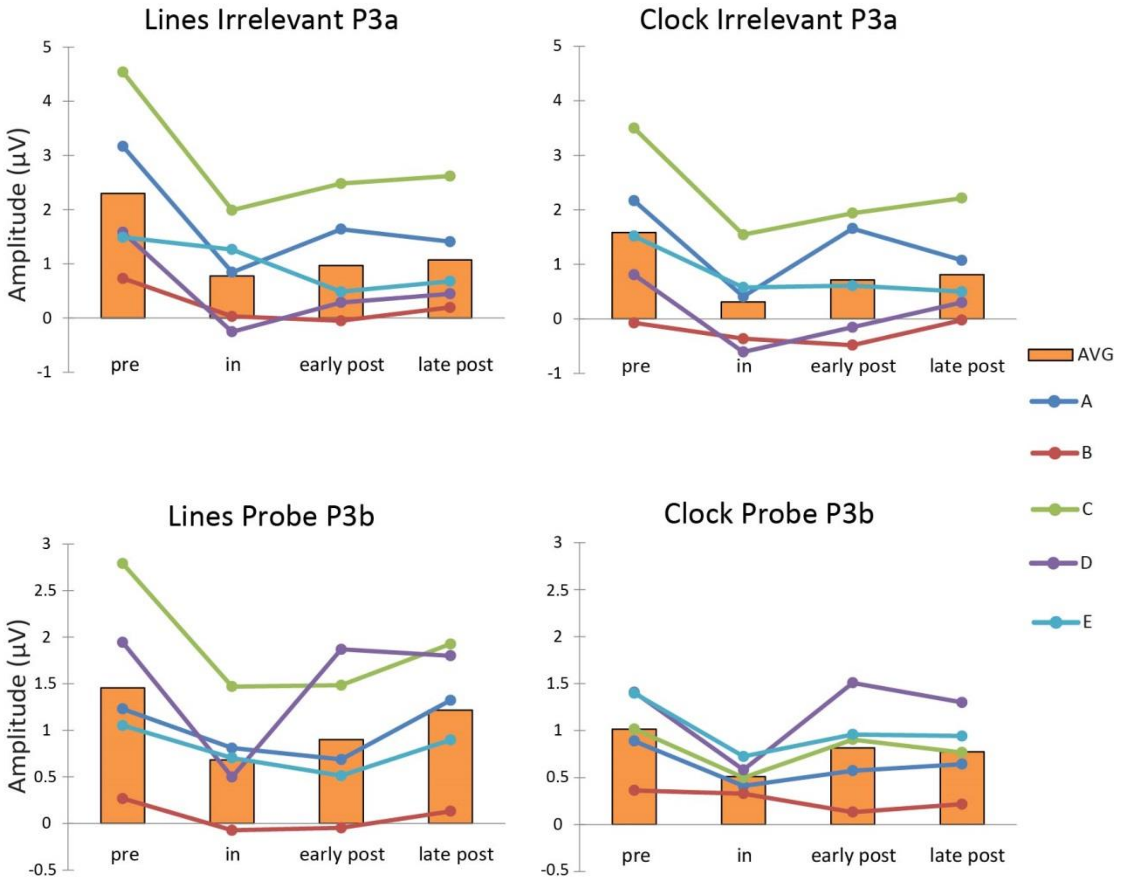

Supplementary Figure S9. Mean (AVG) and individual (A, B, C, D and E subjects) ERP amplitudes in the Lines (left) and the Clock (right) tasks over pre-, in-, early post- and late post-flight; Amplitudes are averaged over the analyzed electrode matrix. P3a (upper panels) and P3b (bottom panels)

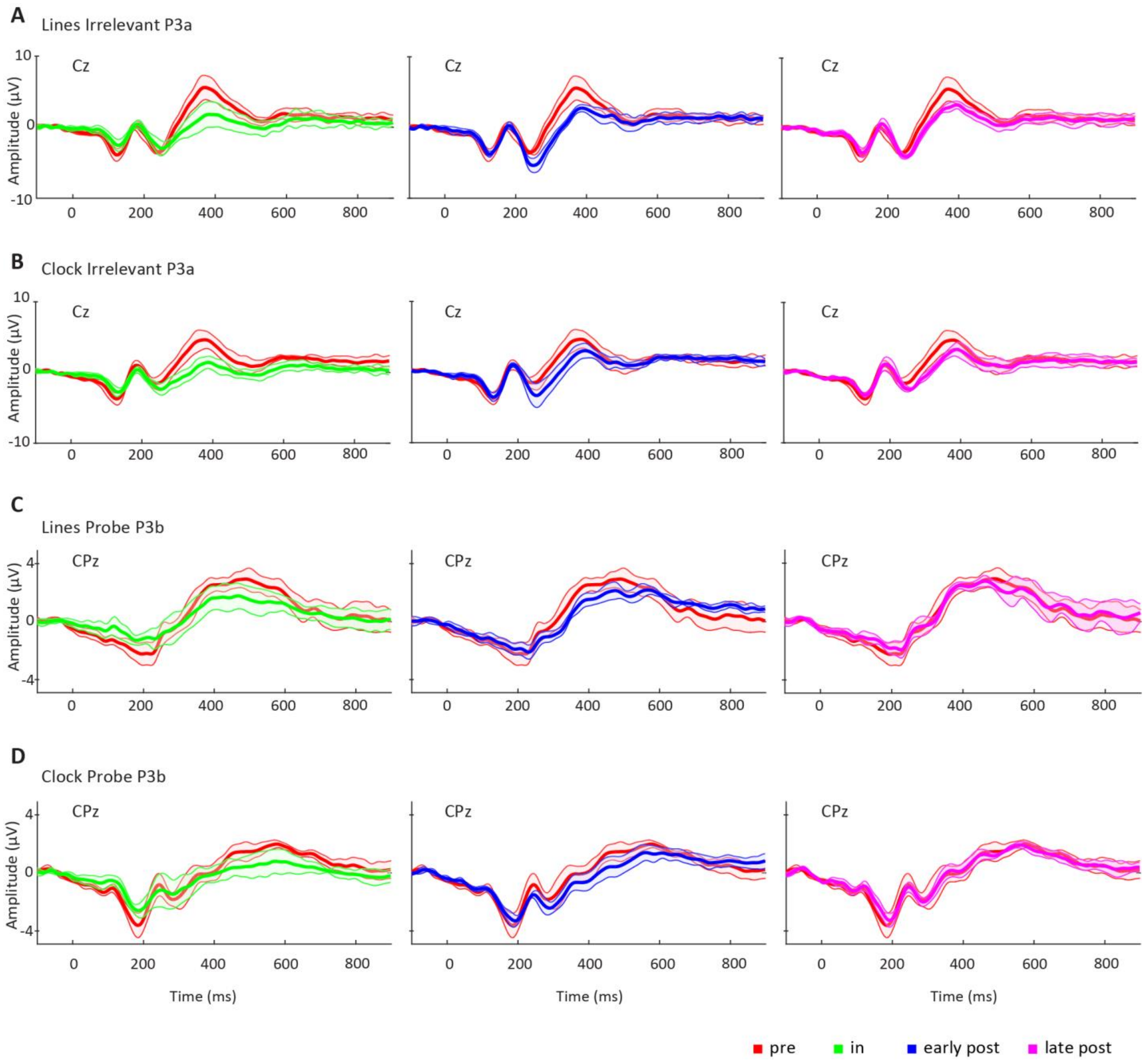

Supplementary Figure S10. Pairwise comparison of event-related potentials in-flight (green lines), early post-flight (blue) and late post-flight (magenta) periods with pre-flight baseline (red lines). Shaded areas represent confidence intervals (alfa=0.05) based on within subjects standard errors [2] in the Lines and the Clock task.

Upper panels: Grand mean ERP at Cz electrode elicited by Irrelevant stimuli in the Lines (A) and Clock (B) task

Lower panels: Grand mean ERP at CPz electrode elicited by Probe stimuli in the Lines (C) and Clock (D) task

Visual frame effect on P3a and P3b

To investigate the visual frame effect regarding P3a and P3b, two separate four-factor rANOVAs [Session (1:9) × Visual Frame (Frame, NoFrame) × Region (Frontal, Central, Parietal) × Laterality (Left, Midline, Right)] were calculated (Supplementary Fig. S11 and S12). No significant main effect of Visual Frame [P3a: $F(1,4) = 0.22$, $P = 0.66$, $\eta_p^2 = 0.05$; P3b: $F(1,4) = 0.04$, $P = 0.85$, $\eta_p^2 = 0.01$] and no interactions between Visual Frame and Session [P3a: $F(8,32) = 3.57$, $P = 0.07$, $\eta_p^2 = 0.47$; P3b: $F(8,32) =$

0.37, P = 0.76, η_p^2 = 0.08], Visual Frame and Region [P3a: F(2,8) = 0.04, P = 0.88, η_p^2 = 0.01; P3b: F(3,12) = 0.21, P = 0.68, η_p^2 = 0.05] or Visual Frame and Laterality [P3a: F(2,8) = 0.21, P = 0.81, η_p^2 = 0.05; P3b: F(2,8) = 1.01, P = 0.40, η_p^2 = 0.20] were identified. Neither of the planned contrasts revealed any significant spaceflight related changes regarding the frame effect (Supplementary Table S3).

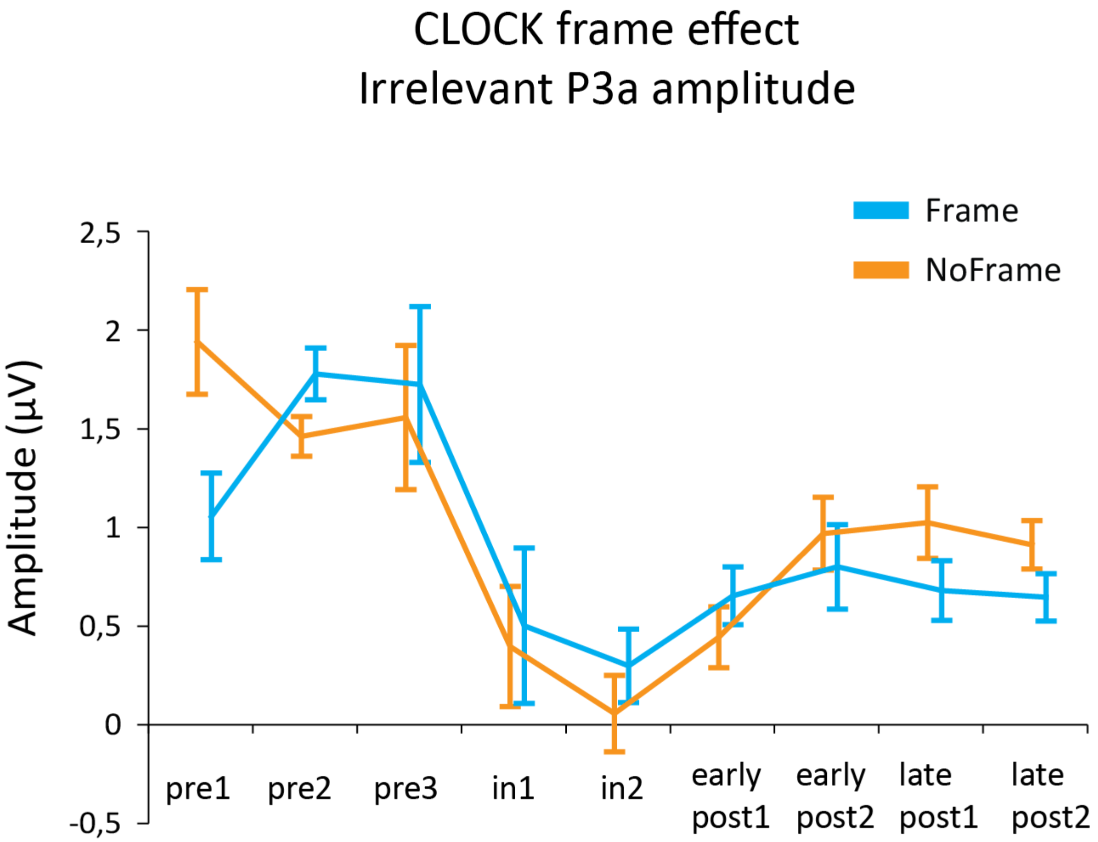

Supplementary Figure S11. Mean amplitude of P3a elicited by Irrelevant stimuli in the Clock task in Frame and NoFrame conditions over all 9 sessions. Amplitudes are averaged over the analyzed electrode matrix. Error bars denote within-subjects SEM [2].

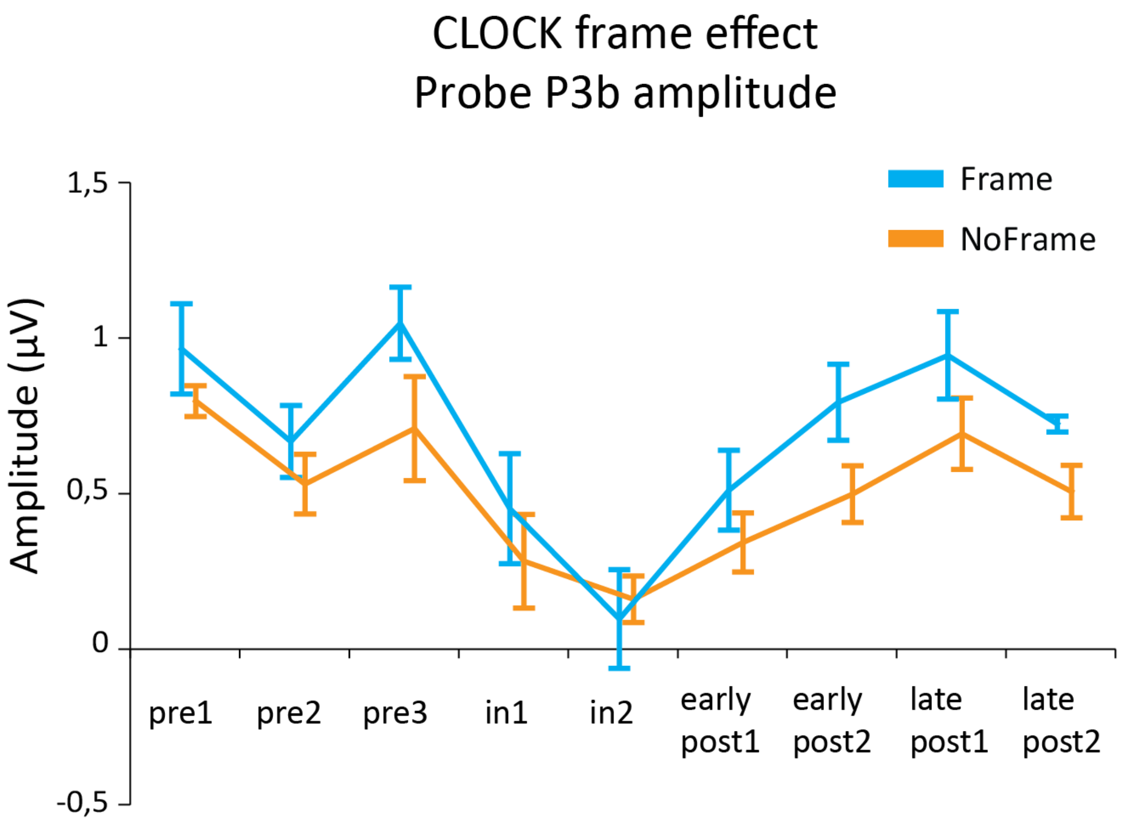

Supplementary Figure S12. Mean amplitude of P3b elicited by Probe stimuli in the Clock task in Frame and NoFrame conditions over all 9 sessions. Amplitudes are averaged over the analyzed electrode matrix. Error bars denote within-subjects SEM [2].

Sleep and fatigue

Data are presented in Supplementary Table S4. Sleep quality was similar across time periods ($\chi2(8)$ = 9.65, P = 0.29). Astronauts reported similar number of nighttime awakenings ($\chi2(8)$ = 7.07, P = 0.53), and same level of morning ($\chi2(8)$ = 6.55, P = 0.59) and daytime sleepiness ($\chi2(8)$ = 5.40, P = 0.71) and daytime fatigue ($\chi2(8)$ = 3.96, P = 0.86) across the experiment.

Supplementary Table S4. Average (±SD) sleep quality, and measures of subjective fatigue and sleepiness.

	**pre-flight1**	**pre-flight2**	**pre-flight3**	**in-flight1**	**in-flight2**	**early post-flight1**	**early post-flight2**	**late post-flight1**	**late post-flight2**
Sleep quality (0-3)	1.4 (±0.55)	1.2 (±0.45)	1.8 (±0.84)	2 (±1.22)	1.2 (±0.45)	1.6 (±0.55)	1.6 (±0.55)	1 (±0.71)	1.2 (±0.84)
Number of nighttime awakenings (0-4)	1 (±1.22)	0.4 (±0.55)	1.2 (±1.64)	1.6 (±1.52)	1 (±1)	1.4 (±1.14)	1.8 (±0.84)	1 (±1.73)	0.8 (±1.1)
Morning sleepiness (0-1)	0.4 (±0.55)	0 (±0)	0.6 (±0.55)	0.4 (±0.55)	0.4 (±0.55)	0.2 (±0.45)	0.4 (±0.55)	0.4 (±0.55)	0.2 (±0.45)
Daytime sleepiness (0-1)	0.6 (±0.55)	0.2 (±0.45)	0.6 (±0.55)	0.8 (±0.45)	0.6 (±0.55)	0.6 (±0.55)	0.6 (±0.55)	0.4 (±0.55)	0.4 (±0.55)
Daytime fatigue (0-3)	1.4 (±0.55)	1.4 (±0.89)	1.2 (±1.1)	1.4 (±0.89)	1.8 (±0.84)	1.8 (±0.84)	1.4 (±0.55)	1.2 (±0.84)	1.4 (±1.14)

Higher values represent worse sleep quality, more awakenings, increased sleepiness and fatigue.

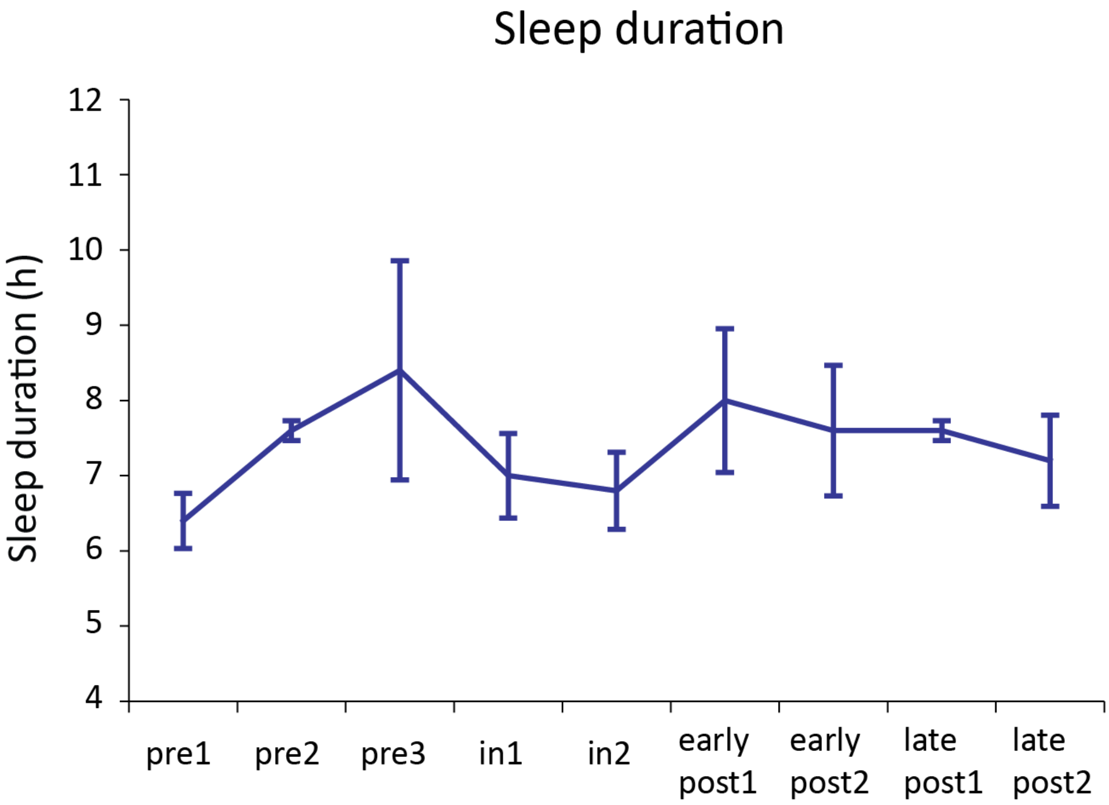

Supplementary Figure S13. Sleep duration over all 9 sessions. Error bars denote within-subjects SEM [2].

References

1. Newell, A. & Rosenbloom, P. S. Mechanisms of skill acquisition and the law of practice. in Cognitive Skills and Their Acquisition (ed Anderson, J. R.) 1-55 (NJ: Erlbaum, Hillsdale, 1981). doi:10.1.1.910.5264.

2. Morey, R. D. Confidence Intervals from Normalized Data: A correction to Cousineau (2005). TQMP, 4, 61–64 (2008). doi:10.20982/tqmp.04.2.p061
